# Síndrome de Klinefelter con deleción del brazo largo del cromosoma X

**DOI:** 10.1515/almed-2022-0076

**Published:** 2022-12-06

**Authors:** Vanesa Escribano Hernández, Mᵃ Ángeles Sanz de Benito, Raffaelle Carraro Casieri

**Affiliations:** Servicio de Análisis Clínicos, Hospital Universitario de La Princesa, Madrid, España; Servicio de Endocrinología y Nutrición, Hospital Universitario de La Princesa, Madrid, España

**Keywords:** deleción, hipogonadismno, síndrome de Klinefelter

Estimado Editor,

El síndrome de Klinefelter (SK) fue descrito por primera vez por Harry F. Klinefelter en 1.942 en 9 hombres con anormalidades testiculares, fallo en la producción de esperma y ginecomastia [1], constituyendo la primera anomalía de los cromosomas sexuales descrita. En 1.959 Jacobs y Strong descubrieron que este fenotipo se debía a la presencia de un cromosoma X adicional [2].

Se trata de una aneuploidía que afecta a los cromosomas sexuales, caracterizada clásicamente por la presencia de dos cromosomas X y un cromosoma Y en individuos que fenotípicamente se corresponden con el sexo masculino. Constituye la anormalidad de los cromosomas sexuales más habitual, con una incidencia aproximada de 1/600 recién nacidos varones, y la causa más frecuente de infertilidad de origen genético en los varones, con una frecuencia del 4% [3]. Clínicamente los pacientes presentan talla alta, hombros estrechos, caderas anchas, debilidad muscular, escaso vello corporal, testículos de pequeño tamaño, ginecomastia e infertilidad. Las pruebas complementarias revelan fallo de la espermatogénesis, niveles disminuidos de testosterona sérica, niveles elevados de las hormonas folículoestimulante (FSH) y luteinizante (LH) y deficiencia androgénica (hipogonadismo hipergonadotropo). Existe mayor asociación con otras patologías como osteoporosis, venas. varicosas, enfermedad tromboembólica, diabetes, bronquitis crónica, bronquiectasias, enfisema y enfermedades neoplásicas. Los rasgos fenotípicos pueden ser tenues y pasar desapercibidos durante la infancia y la adolescencia, llegando al diagnóstico en la etapa puberal por escaso desarrollo de los caracteres sexuales secundarios o incluso en la edad adulta por la presencia de infertilidad.

Presentamos el caso de un varón de 26 años que es derivado a la consulta de urología por fimosis. Refiere pubarquia a los 12 años y cambio de voz y aparición de barba a los 18 años, aunque esta es escasa y se afeita una vez por semana. Tiene erecciones y deseo sexual conservados, y no refiere otros síntomas.

En la exploración física se observa una talla de 177 cm, peso 76,6 kg y envergadura de 180 cm. Presenta leve fimosis, testes hipotróficos y ligera ginecomastia.

En el seminograma se obtiene un volumen de esperma de 3,2 mL, de aspecto translúcido, licuefacción total a la hora y viscosidad normal, con pH de 8,5. Se observa azoospermia.

En la ecografía escrotal se encuentra disminución del tamaño testicular, hipogenicidad difusa y puntos hiperecogénicos en relación con microcalcificaciones, sin lesiones intraparenquimatosas. Epidídimos de morfología conservada con quiste de 8 mm en cabeza de epidídimo izquierdo. No se aprecian hidrocele ni varicocele.

Los hallazgos analíticos del perfil hormonal se exponen en la Tabla 1.

**Tabla 1:** Perfiles hormonales del paciente al diagnóstico.

	20/05/2016	19/10/2016	Intervalo referencia	Unidades
LH	13,46	13,75	0,57–12,07	mUI/mL
FSH	33,23	28,63	0,95–11,95	mUI/mL
PRL	25,93	24,35	3.46,19.40	ng/mL
Estradiol		14	11–44	pg/mL
Progesterona		0,3	0,1–0,2	ng/mL
Testosterona	1,99	1,83	2,40–8,70	ng/mL
Testosterona libre calculada		146,8	228–720	pmol/L
Índice androgénico		27,95		
SHBG		22,7	14,5–48,4	nmol/L

LH, hormona luteinizante; FSH, hormona folículoestimulante; SHBG, globulina fijadora de hormonas sexuales. Cálculo de testosterona libre: fórmula de Vermeulen. Cálculo del índice androgénico: [Testosterona total (nmol/L)/SHBG (nmol/L)] × 100.

Con el diagnóstico de hipogonadismo hipergonadotropo primario (testosterona disminuida con LH y FSH aumentadas) se deriva a la consulta de genética, donde se solicita estudio citogenético encontrándose en todas las metafases observan 47 cromosomas con fórmula sexual XXY y deleción subcentromérica del brazo largo de cromosoma X. Se realiza hibridación genómica comparada (array CGH) con un control masculino (Promega) sobre la plataforma KaryoArray^®^v3.0 (Agilent), con una resolución media de 1 oligo cada 9 kb en las regiones de máximo interés (detección de pérdidas o ganancias de más de 25 kb) y de 175 kb en el resto (detección de pérdidas o ganancias de más de 400 kb). El resultado es un patrón genómico correspondiente a sexo masculino y fórmula arr [hg19] Xp22.33 q13.3 (60,679–74,057,587) × 2. Se encuentran 2 copias de un intervalo aproximado de 74 Mb localizado en el cromosoma X región p.22.33-q13.3, siendo el número total de copias en individuos varones 1. Los hallazgos encontrados en el estudio genético explican el fenotipo que presenta el paciente.

El paciente realiza seguimiento en la consulta de endocrinología, donde pautan sustitución androgénica.

Alrededor del 80% de los pacientes con SK presentan un cariotipo 47,XXY. De ellos, cerca del 50% deben el cromosoma X extra al padre y el otro 50% a la madre. Cuando se debe a herencia materna, el error se produce durante las meiosis I o II o en la división mitótica temprana del desarrollo embrionario. Por el contrario, en las aberraciones debidas a herencia paterna el error sucede exclusivamente en la meiosis. El 20% restante tienen otras alteraciones numéricas de los cromosomas sexuales (48,XXXY; 48,XXYY; 49,XXXXY), mosaicismos 46,XY/47XXY o alteraciones estructurales del cromosoma X [4].

Existen en la literatura pocos casos descritos de pacientes con SK que presenten anormalidades en uno de los cromosomas X. Frühmesser et al. [5] recogen 5 casos de la bibliografía con deleciones en uno de los cromosomas X de los pacientes. Posteriormente en el año 2.015 Liang et al. [6] publicaron un caso varón de 24 años con infertilidad como síntoma inicial, en el que los estudios genéticos revelaron una aneuploidía asociada a SK debida a un cromosoma X extra derivado de herencia materna. Las características fenotípicas y genotipos de los pacientes descritos en la literatura y el del caso que nos ocupa se resumen en la Tabla 2.

**Tabla 2:** Cariotipo y características fenotípicas de pacientes con SK y deleción cromosoma X descritos por Frühmesser et al. y Liang et al. y caso actual.

Autor	Cariotipo	Edad	Altura, cm	Peso, kg	Ginecomastia	Hipotrofia testicular	FSH	LH	Testosterona
Nielsen 1.966	47,XY,der(X)[63]/46,XY [6]	54	160	–	No	SÍ	–	–	–
Chandra et al. 1.971	47,XXq-Y	–	Alto	–	SÍ	SÍ	–	–	–
Nielsen 1.976	47,X,del(X) (p11→q13:q21→q24),del(Y) (q11)	18	163.5	52,9	No	SÍ	↑	↑	Normal
Patil et al. 1.981	47,XY,del(X) (pter→q22:)	19	181	67,5	SÍ	SÍ	↑	↑	↓
Fryns 1.981	47,XY,+del (Xp11)	30	172	59	No	SÍ	↑	Normal	↓
Lian et al. 2.015	47,XY,+der(X)t (X;18) (p11.4;p11.22)	24	179	66	–	SÍ	↑	↑	↓
Caso actual	47,XXY,del (X) (q13.3;qter)	26	177	76,6	SÍ	SÍ	↑	↑	↓
